# dipm: an R package implementing the Depth Importance in Precision Medicine (DIPM) tree and Forest-based method

**DOI:** 10.1093/bioadv/vbac041

**Published:** 2022-06-13

**Authors:** Victoria Chen, Cai Li, Heping Zhang

**Affiliations:** Department of Biostatistics, Yale University, New Haven, CT 06520, USA; Department of Biostatistics, St. Jude Children’s Research Hospital, Memphis, TN 38105, USA; Department of Biostatistics, Yale University, New Haven, CT 06520, USA

## Abstract

**Summary:**

The Depth Importance in Precision Medicine (DIPM) method is a classification tree designed for the identification of subgroups relevant to the precision medicine setting. In this setting, a relevant subgroup is a subgroup in which subjects perform either especially well or poorly with a particular treatment assignment. Herein, we introduce, dipm, a novel R package that implements the DIPM method using R code that calls a program in C.

**Availability and implementation:**

dipm is available under a GPL-3 licence on CRAN https://cran.r-project.org/web/packages/dipm/index.html and at https://ysph.yale.edu/c2s2/software/dipm. It is continuously being developed at https://github.com/chenvict/dipm.

**Supplementary information:**

Supplementary data are available at *Bioinformatics Advances* online.

## 1 Introduction

In recent years, there has been a shift in medicine toward the more modern approach known as precision medicine (Ashley, 2016). The traditional evidence-based medicine collects data from meta-analyses and randomized controlled trials, from which mean estimates are derived to infer general recommendations, approximating the ‘one size fits all’ scenario (Beckmann and Lew, 2016). Precision medicine diverges from the traditional focus on average treatment effects and instead considers what the optimal treatment is for each individual. Moving toward a more targeted approach takes into greater consideration the heterogeneity that exists in patient populations. Overall, the aim of precision medicine is to better deliver safe and effective treatments to patients by identifying the best treatment for each individual. The Depth Importance in Precision Medicine (DIPM) method is a biostatistical approach to realizing the aims of precision medicine (Chen and Zhang, 2020, 2022). The DIPM method is a classification tree method designed to identify subgroups of patients that perform especially well or especially poorly with a particular treatment assignment. Currently, the DIPM method is built for the analysis of clinical datasets with either a continuous (Chen and Zhang, 2020) or right-censored survival outcome (Chen and Zhang, 2022) and two or more treatment groups. Candidate split variables supplied by the user are mined by the method in search of the most important ones. Motivated by the work done by Chen *et al.* (2007) and Zhu *et al.* (2017), the DIPM method uses a depth variable importance score to assess the importance of each candidate split variable at each node of the tree. Chen and Zhang (2022) applied the DIPM method to analyze a microarray dataset for breast cancer patients and identified new gene expression subgroups that are statistically meaningful. We have developed the dipm R package, which implements the DIPM method in addition to a method simpler in design with the same research aims. In this application note, we present an overview of the package and illustrate the usage of dipm through real datasets. The Supplementary Material contains a manual (the vignette for the package).

## 2 Methods and implementation

The DIPM method is designed for the analysis of clinical datasets with either a continuous or right-censored survival outcome variable *Y* and two or more treatment assignments (Chen and Zhang, 2020, 2022). Without loss of generality, higher values of *Y* denote better health outcomes. Note that this is also true for the survival case only when the event of interest is harmful, as longer times to the harmful event are more beneficial. When *Y* is a right-censored survival outcome, the data must also contain a status indicator *δ*. When *δ* = 1, this indicates that the event of interest has occurred, while *δ* = 0 indicates that an observation is right-censored.

**Fig. 1.**
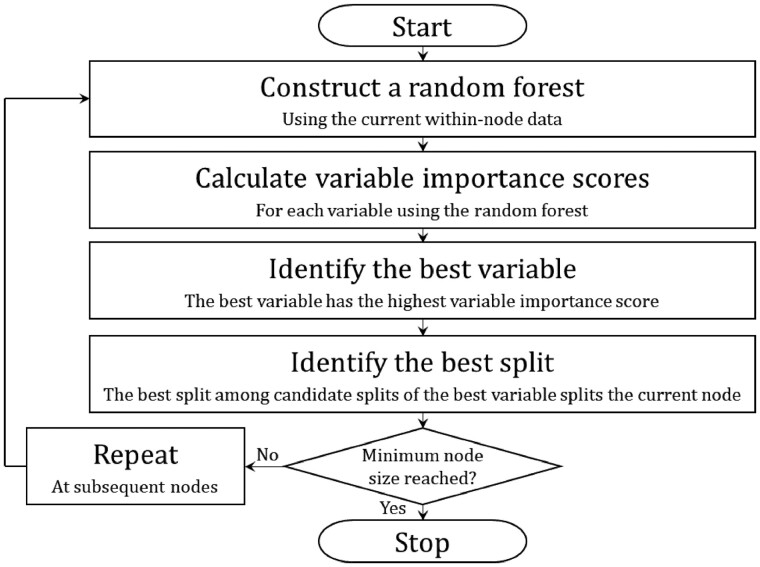
Overview of DIPM method classification tree algorithm. A flowchart outlining the general steps of the proposed method’s algorithm is depicted in the figure above

Candidate split variables are also part of the data and may be binary, ordinal or nominal. All of the learning data are said to be in the first or root node of the classification tree, and nodes may be split into two child nodes. Borrowing the terminology used in Zhu *et al.* (2017), at each node in the tree, a random forest of ‘embedded’ trees is grown to determine the best variable to split the node. Once the best variable is identified, the best split of the best variable is identified based on a calculated score. A flowchart outlining the general steps of the DIPM algorithm is provided in Figure 1.

In the DIPM method, the depth variable importance score is used to find the best split variable at a node. The score is a relatively simple measure that takes into account solely two components: the depth of a node within a tree and the magnitude of the relevant effect. Using depth information makes use of the observation that more important variables tend to be selected closer to root nodes of trees. Meanwhile, the strength of the split is also taken into account. This second component is a statistic specified depending on the particular analysis and data at hand. Recall that at each node in the overall classification tree, a random forest is constructed to find the best split variable at the node. Once the forest is fit, for each tree *T* in this forest, the following sum is calculated for each covariate *j*:

(1) $$\mathrm{score}(T,j)=\sum_{t\in T_{j}}2^{-L(t)}G_{t}.$$

*T_j_* is the set of nodes in tree *T* split by variable *j*. *L*(*t*) is the depth of node *t*. For example, the root node has depth 1, the left and right child nodes of the root node have depth 2. *G_t_* captures the magnitude of the effect of splitting node *t*. Depending on the type of data available, the test statistic *G_t_* will vary. Next, the split criteria used are defined depending on the type of outcome variable and the number of treatment assignments as well. See the Supplementary Material for more details.

The dipm package contains two main functions: dipm and spmtree. The dipm function generates classification trees for the precision medicine setting as described above. The spmtree is also designed for the same aim as a simpler tree method. However, this method does not fit a random forest at each node. Instead, the more classical approach of considering all possible splits of all candidate split variables is used, and the single split with the highest split criteria score is selected as the ‘best’ split of the node. For each method, the R code calls a C program to generate each tree. The C backend is used to take advantage of C’s higher computational speed in comparison to R. Furthermore, the R package has been designed to remain consistent with existing R package implementations of tree-based methods such as rpart (Therneau and Atkinson, 2018) and partykit (Hothorn and Zeileis, 2015). Maintaining consistent function arguments across packages is helpful so that users can focus on the analysis at hand instead of spending excessive amounts of time deciphering the intricacies unique to each package. In addition, the package contains a pruning function pmprune that removes terminal sister nodes with the same optimal treatment. This package also contains the function node_dipm, which is specially designed for subgroup analysis and compatible with the plot method defined in the partykit package. It visualizes stratified treatment groups through boxplots for a continuous outcome and survival plots for a survival outcome, respectively.

## 3 Example usage

For both the dipm and spmtree functions, at a minimum, the user must supply a formula and a dataset. For the formula argument, the formula must take format Y ∼ treatment | X1 + X2 for data with a continuous outcome variable *Y* and Surv(Y, *δ*) ∼ treatment | X1 + X2 for data with a survival outcome variable *Y* and a status indicator *δ*. A format such as Y ∼ treatment |. may be used when all variables in the data, excluding *Y*, *δ* (if applicable), and the treatment variable, are to be used as candidate split variables. For the data argument, the supplied dataset must contain an outcome variable *Y* and a treatment variable. If *Y* is a right-censored survival time outcome, then there must also be a status indicator *δ*. The types argument is optional. When the types argument is missing, the default is to assume all of the candidate split variables are ordinal, which includes numeric variables. If this is not the case, then all of the variables in the data must be specified with a vector of characters in the order that the variables appear. The possible variable types are: ‘binary’, ‘ordinal’, ‘nominal’, ‘response’, ‘status’ and ‘treatment’. Detailed instructions, examples and returned output can be found in the Supplementary Material.

**Fig. 2.**
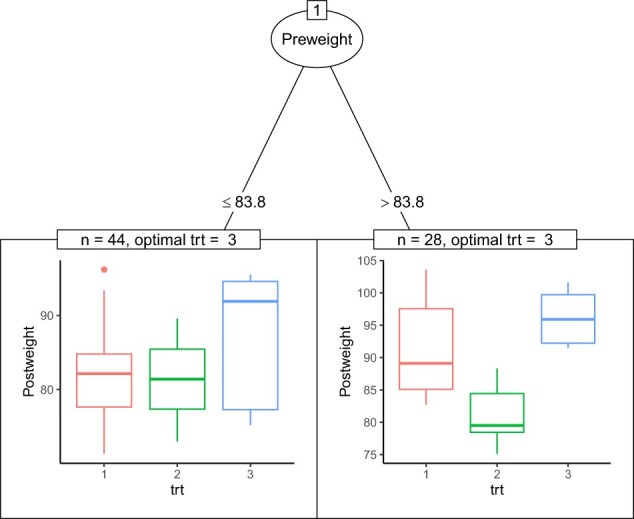
Tree visualization of anorexia data using function node_dipm

The weight change data (MASS::anorexia) for young female anorexia patients from the MASS package consists of 72 observations and 3 variables (Venables and Ripley, 2013). The data contain three treatment groups: (i) for cognitive behavioral treatment, (ii) for control and (iii) for family treatment. PreWeight is the weight in pounds of the patient before study period. Similarly, PostWeight represents that of the patient after study period. In our analysis, we consider PostWeight as the response of interest and fit a tree based on the DIPM method. Figure 2 visualizes the tree using the function node_dipm. For both identified subgroups, family treatment is identified as the optimal treatment. However, the effect of cognitive behavioral treatment versus control is more profound in the subgroup of patients with higher weights before the study than in the subgroup of patients with lower weights before the study.

**Fig. 3.**
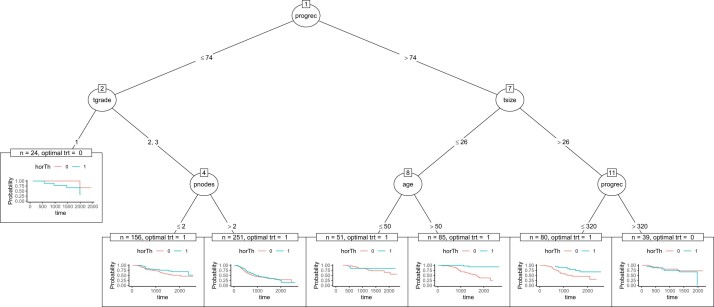
Tree visualization of breast cancer data using function node_dipm

The dataset (TH.data::GBSG2) from the package TH.data contains the observations of 686 women from the German Breast Cancer Study Group (Sauerbrei *et al.*, 2000). The treatment is hormonal therapy (0 for no, 1 for yes). The detailed descriptions of other variables can be seen in the Supplementary Material. We fit a survival tree based on the DIPM method and visualize the tree using the function node_dipm in Figure 3. Hormonal therapy is most effective except for the patients with progesterone receptor less than 74 fmol and tumor grade I or progesterone receptor more than 74 fmol and tumor size greater than 26 mm and progesterone receptor greater than 320 fmol.

## 4 Significance and conclusions

In summary, the dipm R package implements the DIPM classification tree method designed for the analysis of clinical datasets with a continuous or right-censored survival outcome variable and two or more treatment groups. A secondary, additional method is also included in the package that employs a much simpler approach in identifying the best split at a node. Both methods have been carefully evaluated in previous works (Chen and Zhang, 2020, 2022). Furthermore, we develop a plotting function that produces an image of each tree instead of solely a data frame of nodes. Overall, this package delivers a new and handy computational tool that implements the novel DIPM method in the search for subgroups relevant to the precision medicine setting.

## Funding

This work was supported in part by the National Institutes of Health [R01HG010171 and R01MH116527] and the National Science Foundation [DMS1722544 and DMS2112711].

*Conflict of Interest*: none declared.

## Supplementary Material

vbac041_Supplementary_Data
