# Supplementary material for: dipm: an R package implementing the Depth Importance in Precision Medicine (DIPM) tree and Forest-based method
Source: Bioinform Adv. 2022 Jun 13;2(1):vbac041. doi: 10.1093/bioadv/vbac041 (PMC9245626; doi:10.1093/bioadv/vbac041)
Supplement: vbac041_Supplementary_Data [file vbac041_supplementary_data.pdf]

dipm: An R Package Implementing the Depth Importance in Precision Medicine (DIPM) Tree and Forest Based Method

Victoria Chen*
Yale University

Cai Li*
St. Jude Children's Research Hospital

Heping Zhang
Yale University

Abstract

The Depth Importance in Precision Medicine (DIPM) method is a classification tree designed for the identification of subgroups relevant to the precision medicine setting. In this setting, a relevant subgroup is a subgroup in which subjects perform either especially well or poorly with a particular treatment assignment. The **dipm** R package implements the DIPM method using R code that calls a program in C. Overall, the DIPM method is built to analyze clinical datasets with either a continuous or right-censored survival outcome variable and two or more treatment groups. The data also contain a number of candidate split variables supplied by the user. Within the method, the candidate split variable with the largest depth variable importance score is identified as the best variable to split the node. An additional simpler tree method that does not fit a random forest at each node is also included in the package. All of the functions in the package are explained, and illustrative examples are provided to help guide anyone aiming to use the DIPM method with the analysis of datasets of their own.

Keywords: binary tree, classification tree, continuous outcomes, dipm, precision medicine, R, random forest, right-censored, subgroup analysis, survival outcomes, variable importance.

1. Introduction

In recent years, there has been a shift in medicine towards the more modern approach known as precision medicine (Ashley 2016). Precision medicine diverges from the traditional focus on average treatment effects and instead considers what the optimal treatment is for each individual. Moving towards a more targeted approach takes into greater consideration the heterogeneity that exists in patient populations. Overall, the aim of precision medicine is to better deliver safe and effective treatments to patients by identifying the best treatment for each individual.

The Depth Importance in Precision Medicine (DIPM) method is a biostatistical approach to realizing the aims of precision medicine (Chen and Zhang 2020, 2022). The DIPM method is a classification tree method designed to identify subgroups of patients that perform especially well or especially poorly with a particular treatment assignment. Currently, the DIPM method is built for the analysis of clinical datasets with either a continuous (Chen and Zhang 2020) or right-censored survival outcome (Chen and Zhang 2022) and two or more treatment groups. Candidate split variables supplied by the user are mined by the method in search of the most

important ones. Motivated by the work done by Chen, Liu, Zhang, and Zhang (2007) and Zhu, Zhao, Chen, Ma, and Zhao (2017), the DIPM method uses a depth variable importance score to assess the importance of each candidate split variable at each node of the tree.

In this vignette, we present the **dipm** R package. The package implements the DIPM method in addition to a method simpler in design with the same research aims. For each method, the R code calls a C program to generate each tree. The C backend is used to take advantage of C's higher computational speed in comparison to R. Furthermore, the R package has been designed to remain consistent with existing R package implementations of tree based methods also created for the precision medicine setting. Maintaining consistent function arguments across packages is helpful so that users can focus on the analysis at hand instead of spending excessive amounts of time deciphering the intricacies unique to each package.

The remainder of this vignette is structured as follows. First, existing R packages with functions that fit trees also designed for the precision medicine are briefly described for comparison. Then, the DIPM method is described in greater detail. Next, the functions and corresponding arguments of the **dipm** R package are explained. Illustrative examples of the **dipm** R package are then provided. Finally, the discussion section presents concluding remarks.

2. Previous R Packages

Currently, there exist R package implementations of tree based methods in general. Consider the popular **rpart** package (Therneau and Atkinson 2018) which fits recursive partitioning and regression trees. Another example is the **randomForest** package (Liaw and Wiener 2002) which fits a random forest of trees using Breiman's algorithm. Though our **dipm** R package implements our novel tree based method for precision medicine, there are other existing R packages which implement tree based methods also designed specifically for the precision medicine setting. These existing packages include: **party** (Zeileis, Hothorn, and Hornik 2008; Hothorn, Hornik, and Zeileis 2006) and its successor **partykit** (Hothorn and Zeileis 2015; Seibold, Zeileis, and Hothorn 2016), **quint** (Dusseldorp and Van Mechelen 2014; Dusseldorp, Doove, van de Put, and Van Mechelen 2018), **RLT** (Zhu *et al.* 2017), and **model4you** (Seibold, Zeileis, and Hothorn 2019).

Within the **party** and **partykit** R packages, the `mob()` function fits a tree using the model-based recursive partitioning tree method developed by Zeileis *et al.* (2008). This method is able to analyze data with either a continuous or right-censored survival outcome variable. Next, within the **quint** R package, the `quint()` function fits QUalitative INteraction Trees for subgroup analysis as developed by Dusseldorp and Van Mechelen (2014). The `quint()` function is designed for the analysis of clinical data with two treatment arms and a continuous outcome variable. Next, within the **RLT** R package, the `RLT()` function may be used to fit an importance index based classification tree method developed by Zhu *et al.* (2017). This method is designed for the analysis of data with two treatment assignments and a continuous outcome variable. However, in their paper, Zhu *et al.* (2017) describe how the `RLT()` function may also be used for data with right-censored survival outcomes and/or more than two treatment assignments. Lastly, within the **model4you** R package, the `pmtree()` function fits a model-based tree using a linear regression or cox regression model fit. This method is designed for scenarios where two treatments are compared and responses of interest possibly depend on other variables.

Since the **partykit**, **quint**, and even **rpart** and **randomForest** R packages use a formula argument to specify the outcome and candidate split variables of a supplied dataset, we remain consistent with these packages by also using a formula argument. Since tree based methods designed for the precision medicine setting have the unique feature of requiring a treatment variable, we specifically remain consistent with the notation used in both the **partykit** and **quint** R packages by using notation such as `Y ~ treatment | X1 + X2`. More details about the notation required for the formula argument may be found in Section 4.5 below. Though the method by Zhu *et al.* (2017) is more similar to our DIPM method, their `RLT()` function deviates from existing methods and does not use a formula argument. Instead, candidate split variables are specified as a matrix or data frame in a separate `X` argument, the treatment variable is specified as a vector in its own `y` argument, and the response variable information is captured in a vector of weights that are specified in the `subject.weight` argument.

As a whole, the **DIPM** R package is designed to be a user-friendly implementation of a tree based method in precision medicine. We prioritize having clear documentation in addition to arguments that are consistent with existing methods so that users may spend less time studying arguments and notation and more time getting to the analysis at hand. The DIPM method also aims to have a broader scope in regard to the types of data that are able to be analyzed, i.e., data with a continuous or right-censored survival outcome variable and two or more possible treatment groups. Also, the method has been designed to have minimal to no required pre-processing steps particularly in comparison with the `RLT()` function. More details about the DIPM method are provided in Section 3 below.

3. Methods

3.1. Overview

The DIPM method is designed for the analysis of clinical datasets with either a continuous or right-censored survival outcome variable Y and two or more treatment assignments (Chen and Zhang 2020, 2022). Without loss of generality, higher values of Y denote better health outcomes. Note that this is also true for the survival case only when the event of interest is harmful, as longer times to the harmful event are more beneficial. When Y is a right-censored survival outcome, the data must also contain a status indicator δ. When $\delta = 1$, this indicates that the event of interest has occurred, while $\delta = 0$ indicates that an observation is right-censored.

Candidate split variables are also part of the data and may be binary, ordinal, or nominal. All of the learning data are said to be in the first or root node of the classification tree, and nodes may be split into two child nodes. Borrowing the terminology used in Zhu *et al.* (2017), at each node in the tree, a random forest of "embedded" trees is grown to determine the best variable to split the node. Once the best variable is identified, the best split of the best variable is identified based on a calculated score. The "best variable" is "best" in a narrow sense as defined below. A flowchart outlining the general steps of the DIPM algorithm is provided in Figure 1.

In addition to the DIPM method, there is a second tree method included in the **dipm** R package. This method is also designed for the analysis of clinical datasets with either a continuous or right-censored survival outcome variable Y and two or more treatment assignments. How-

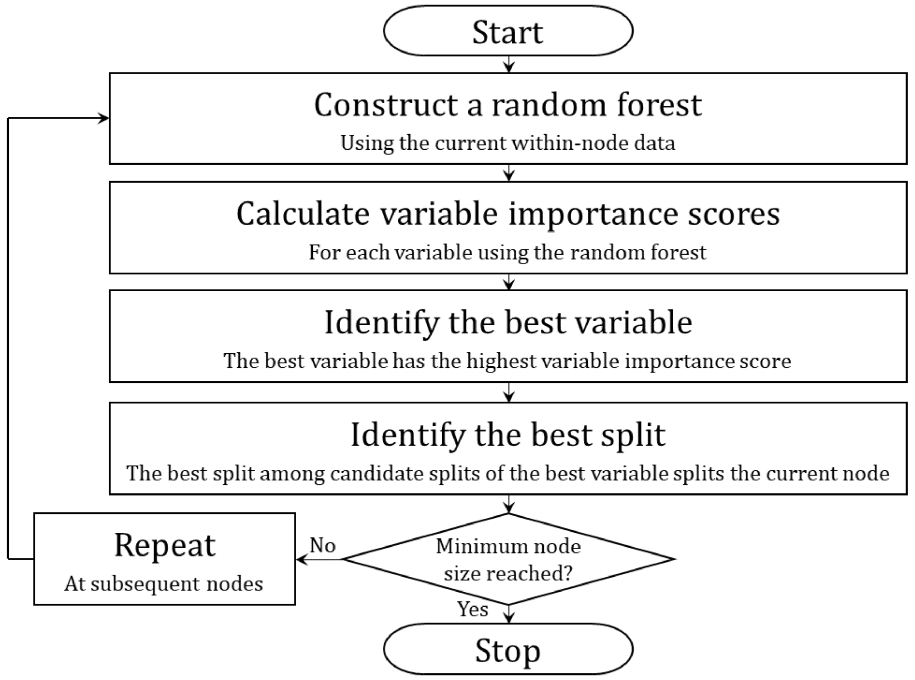

Figure 1: Overview of DIPM method classification tree algorithm. A flowchart outlining the general steps of the proposed method's algorithm is depicted in the figure above.

ever, this method does not fit a random forest at each node. Instead, the more classical approach of considering all possible splits of all candidate split variables is used, and the single split with the highest split criteria score is selected as the "best" split of the node. The specific split criteria are described below. Note that this method is "novel" in the sense that we have implemented the method ourselves, but the general approach is simple and previously established. This simpler method is referred to as the simple precision medicine (SPM) tree method throughout this vignette.

3.2. Depth Variable Importance Score

In the DIPM method, the depth variable importance score is used to find the best split variable at a node. The score is a relatively simple measure that takes into account solely two components: the depth of a node within a tree and the magnitude of the relevant effect. Using depth information makes use of the observation that more important variables tend to be selected closer to root nodes of trees. Meanwhile, the strength of the split is also taken into account. This second component is a statistic specified depending on the particular analysis and data at hand.

Recall that at each node in the overall classification tree, a random forest is constructed to find the best split variable at the node. Once the forest is fit, for each tree T in this forest, the following sum is calculated for each covariate j:

$$score(T, j) = \sum_{t \in T_j} 2^{-L(t)} G_t. \tag{1}$$

T_j is the set of nodes in tree T split by variable j. $L(t)$ is the depth of node t. The root node

has depth 1, the left and right child nodes of the root node have depth 2, etc. G_t captures the magnitude of the effect of splitting node t.

Depending on the type of data available, the test statistic G_t will vary. In particular, we classify the DIPM method into four possible scenarios: (1) datasets with a continuous outcome variable and two treatment groups, (2) datasets with a survival outcome variable and two treatment groups, (3) datasets with a continuous outcome variable and more than two treatment groups, and (4) datasets with a survival outcome variable and more than two treatment groups. For each scenario, note that the specified model is fit using the pertinent within-node data. In each case, the test statistic is squared because the magnitude of the interaction is of interest, while there is no preference in the effect's direction.

For data with a continuous outcome variable and two treatment assignments, G_t is set equal to the t^2 statistic from testing the significance of β_3 in the model:

$$Y = \beta_0 + \beta_1 * trt + \beta_2 * s + \beta_3 * trt * s + \epsilon. \quad (2)$$

trt denotes the treatment variable, and s represents a particular split. Note that the t^2 statistic is identical to the statistic used at each node split in the interaction tree method (Su, Tsai, Wang, Nickerson, and Li 2009).

For data with a survival outcome variable and two treatment assignments, G_t is set equal to the z^2 statistic from testing the significance of β_3 in the Cox model with hazard function:

$$h(t, trt, s) = h_0(t) * exp\{\beta_1 * trt + \beta_2 * s + \beta_3 * trt * s\}, \quad (3)$$

where $h_0(t)$ is the baseline hazard function.

For data with a continuous outcome and more than two treatment assignments, G_t is set equal to the largest t^2 statistic among the split by treatment interaction terms in a linear regression model. More specifically, for data with k total treatment groups, G_t is set equal to the t^2 test statistic of $H_0 : \beta_i = 0$ using $t = \max_{i \in \{k+1, 2k-1\}} |t_i|$ from the model:

$$Y = \beta_0 + \beta_1 trt_1 + ... + \beta_{k-1} trt_{k-1} + \beta_k s + \beta_{k+1} trt_1 s + ... + \beta_{2k-1} trt_{k-1} s + \epsilon. \quad (4)$$

trt_i is the vector denoting assignment to treatment i, and s represents a particular split.

Finally, for data with a survival outcome variable and more than two treatment assignments, G_t is set equal to the largest z^2 Wald test statistic among the split by treatment interaction terms in a Cox model. More specifically, for data with k total treatment groups, G_t is set equal to the z^2 Wald test statistic of $H_0 : \beta_i = 0$ using $z = \max_{i \in \{k+1, 2k-1\}} |z_i|$ from the Cox model with hazard function:

$$h(t, trt, s) = h_0(t) * exp\{\beta_1 trt_1 + ... + \beta_{k-1} trt_{k-1} + \beta_k s + \beta_{k+1} trt_1 s + ... + \beta_{2k-1} trt_{k-1} s\}, \quad (5)$$

where $h_0(t)$ is the baseline hazard function. trt_i is the vector denoting assignment to treatment i, and s represents a particular split.

Next, a "G replacement" feature is implemented that potentially alters the variable importance scores $score(T, j)$. For each tree T in the forest, the G_t at each split is replaced with the highest G_t value of any of its descendant nodes if this maximum exceeds the value at the current split. This replacement step is performed because a variable that yields a split with a large effect of interest further down in the tree is certainly important even if its importance

is not captured right away. By "looking ahead" at the G_t values of future splits, a variable's importance is reinforced.

Lastly, the final variable importance scores are averaged across all M trees in the forest f:

$$score(f, j) = \frac{1}{M} \sum_{T \in f} score(T, j). \tag{6}$$

The best split variable is the variable with the largest value of $score(f, j)$.

3.3. Split Criteria

Next, the split criteria used are defined depending on the type of outcome variable and the number of treatment assignments. Note that these split criteria are used by both the DIPM method and the simple PM tree method. For data with a continuous outcome variable and more than two treatment assignments, to identify the best split at a node t, the squared difference in response rates between the treatment with the largest mean response and the treatment with the smallest mean response at node t is first assessed:

$$DIFF(t) = (\max_{i \in T} \bar{Y}_{i,t} - \min_{i \in T} \bar{Y}_{i,t})^2. \tag{7}$$

T is the set of all treatments. Then, among the list of candidate splits, only splits with child nodes with at least $nmin$ subjects are considered. Of the splits with a sufficient number of subjects, the best split maximizes the weighted sum of the squared difference in response rates of the child nodes:

$$DIFF(t_L, t_R) = \frac{\sum_{s=\{L,R\}} n_s (\max_{i \in T} \bar{Y}_{i,t_s} - \min_{i \in T} \bar{Y}_{i,t_s})^2}{n_L + n_R}. \tag{8}$$

L and R denote the left and right child nodes respectively. Node t is split only when the best split yields two child nodes with a greater difference in treatment response rates than at the current node:

$$DIFF(t_L, t_R) > DIFF(t). \tag{9}$$

Splitting stops when there are not enough subjects in any candidate node splits or when no remaining $DIFF(t_L, t_R)$ values exceed $DIFF(t)$. Note that when the data contain a continuous outcome variable and two treatment groups, essentially the same split criteria are used. The above equations may be simplified as follows. In the two treatment case, denoting the two treatments with A and B, $(\bar{Y}_{A,t} - \bar{Y}_{B,t})^2$ is used in place of equation 7, and $\frac{\sum_{s=\{L,R\}} n_s (\bar{Y}_{A,t_s} - \bar{Y}_{B,t_s})^2}{n_L + n_R}$ is used in place of equation 8.

For data with a survival outcome variable and more than two treatment assignments, the best split is the split with the largest z^2 Wald test statistic that tests the significance of the most significant split by treatment interaction term in the Cox model (5). Similarly, for data with a survival outcome variable and two treatment groups, the best split is the split with the largest z^2 Wald test statistic that tests the significance of the single split by treatment interaction term in the Cox model (3). Among the list of candidate splits, only splits with child nodes with at least $nmin$ subjects are considered. At a given node, splitting stops when there are less than $nmin$ subjects in the child nodes of every candidate split.

3.4. Random Forest

In the DIPM method, a random forest is grown at each node in the overall tree and then used to select the best split variable. Once this variable is identified, all possible splits of the variable are considered, and the best split is found using the criteria described in Section 3.3. The forest contains a total of M embedded trees. Each embedded tree is grown using a bootstrap sample. At each node in the embedded trees, either: (1) all possible splits of all of the variables are considered, or (2) all possible splits of $mtry$ randomly selected variables are considered. The best split is again found using the criteria described in Section 3.3.

3.5. Best Predicted Treatment Class

The best predicted treatment class of a node is the treatment group that performs best based on the subjects within the given node. The following calculations of the best predicted treatment class apply to both the DIPM method and the simple PM tree method. For data with a continuous outcome variable, the means of the response values Y are compared by treatment group. Since higher values of Y denote greater benefit for patients, the best predicted treatment at a node is the treatment group i with the largest mean value $\bar{Y}_i$.

For data with survival endpoints, the best predicted treatment class has the largest mean survival time, as estimated by calculating the area under the Kaplan-Meier curve of the respective treatment group. Since larger areas, i.e., larger mean survival estimates, denote better survival rates when the event of interest is negative, the best predicted treatment at a node is the treatment group i with the largest estimated mean survival time $\hat{\mu}_i = \int_0^{\tau_i} \hat{S}_i(t)dt$. τ_i is set equal to the largest observed time in the respective treatment group, and if the largest observed time is censored, then the largest observed time is converted to an event.

4. Arguments of dipm Package Functions

4.1. Overview

The **dipm** package contains two main functions: `dipm()` and `spmtree()`. "dipm" is shorthand for "depth importance in precision medicine" for the DIPM method, and "spmtree" is shorthand for "simple precision medicine tree" for the simple PM tree method. These functions generate classification trees for the precision medicine setting as described in the Methods section above. The default output is a tree object of S3 class '`party`' defined in the R package **partykit** Hothorn and Zeileis (2015). The benefits of treating the tree as a '`party`' object are two-fold: first, it allows us to leverage the added functionality for print/plot/predict methods; second, it permits more compatibility with other R packages and offers utility functions to manipulate the tree. In addition, the package contains a pruning function that removes terminal sister nodes with the same optimal treatment. This function is called `pmprune()` which is shorthand for "precision medicine pruning". This package also contains the function `node_dipm()`, which is specially designed for subgroup analysis and compatible with the plot method defined in the **partykit** package. It visualizes stratified treatment groups through boxplots for a continuous outcome and survival plots for a survival outcome, respectively.

4.2. The `dipm()` Function

The `dipm()` function creates a classification tree using the DIPM method. Overall, the `dipm()` function has the following arguments:

- `formula`: A description of the model to be fit.
- `data`: A matrix or data frame of the data.
- `types`: A vector of the types of each variable in the data.
- `nmin`: An integer specifying the minimum node size of the overall classification tree.
- `nmin2`: An integer specifying the minimum node size of embedded trees.
- `ntree`: An integer specifying the number of embedded trees to construct at each node of the overall classificiation tree.
- `mtry`: An integer specifying the number of candidate split variables to randomly select at each node of embedded trees if applicable.
- `maxdepth`: An integer specifying the maximum depth of the overall classification tree.
- `maxdepth2`: An integer specifying the maximum depth of embedded trees.
- `print`: A boolean (`TRUE`/`FALSE`) value, where `TRUE` prints a more readable version of the final tree to the screen.
- `dataframe`: A boolean (`TRUE`/`FALSE`) value, where `TRUE` returns the final tree as a data frame.
- `prune`: A boolean (`TRUE`/`FALSE`) value, where `TRUE` prunes the final tree using the `pmprune()` function.

At a minimum, the user must supply a formula and a dataset. For more details about the required attributes for the `formula` and `data` argument inputs, refer to Section 4.5 below. The default for `types` is to assume all of the candidate split variables are ordinal. If this is not the case, instructions for how to specify the variable types are also in Section 4.5. The default values of `nmin` and `nmin2` are both set to 5. Arguments `maxdepth` and `maxdepth2` are both optional. These arguments are useful for shortening computation time, but if left blank, the default for both is to grow the full tree until the minimum node size, `nmin` and `nmin2` respectively, is reached. The default value of the `print` argument is `TRUE`. Lastly, the default values of the `dataframe` and `prune` arguments are `FALSE`.

For the `mtry` argument, the default value is Inf. When `mtry` = Inf, all possible splits of all candidate split variables are considered at the nodes of embedded trees. If instead, one would like to implement the version of the DIPM method in which a random subset of `mtry` of the candidate split variables is considered at the nodes of embedded trees, then a recommended value to use for a dataset with p candidate split variables is $floor(\sqrt{p})$. This value is the default value used in the `randomForest` R package implementing Breiman's random forest method for classification. The aim is to use a value that balances the strength of each tree

by being large enough, while minimizing the correlation between trees by being small enough (Breiman 2001).

For the `ntree` argument, the following recommended values are used by default depending on the value of `mtry`. The recommended values for `ntree` are based on `mtry` mainly due to computation time. When `mtry` = Inf, `ntree` = $\min(\max(\sqrt{n}, \sqrt{p}), 1000)$. When `mtry` is an integer smaller than Inf, `ntree` = $\min(\max(n, p), 1000)$. n is the total sample size of the data, and p is the total number of candidate split variables.

4.3. The `spmtree()` Function

The `spmtree()` function creates a classification tree using the simple PM tree method. Overall, the `spmtree()` function has the following arguments:

- `formula`: A description of the model to be fit.
- `data`: A matrix or data frame of the data.
- `types`: A vector of the types of each variable in the data.
- `nmin`: An integer specifying the minimum node size of the overall classification tree.
- `maxdepth`: An integer specifying the maximum depth of the overall classification tree.
- `print`: A boolean (`TRUE`/`FALSE`) value, where `TRUE` prints a more readable version of the final tree to the screen.
- `dataframe`: A boolean (`TRUE`/`FALSE`) value, where `TRUE` returns the final tree as a data frame.
- `prune`: A boolean (`TRUE`/`FALSE`) value, where `TRUE` prunes the final tree using the `pmprune()` function.

At a minimum, the user must supply a formula and a dataset. For more details about the required attributes for the `formula` and `data` argument inputs, refer to Section 4.5 below. The default for `types` is to assume all of the candidate split variables are ordinal. If this is not the case, instructions for how to specify the variable types are also in Section 4.5. The default value of `nmin` is 5. The `maxdepth` argument is optional. This argument is useful for shortening computation time, but if left blank, the default is to grow the full tree until the minimum node size, `nmin`, is reached. The default value of the `print` argument is `TRUE`. Lastly, the default values of the `dataframe` and `prune` arguments are `FALSE`.

4.4. The `pmprune()` Function

The `pmprune()` function prunes a classification tree using the simple pruning strategy first proposed by Zhang, Legro, Zhang, Zhang, Chen, Huang, Casson, Schlaff, Diamond, Krawetz, Coutifaris, Brzyski, Christman, Santoro, and Eisenberg (2010). The approach simply removes terminal sister node pairs that have the same optimal treatment assignment. The `pmprune()` function is designed to be called by the `dipm()` or `spmtree()` function and accepts as an argument the data frame object returned from either one. Then, the `pmprune()` function

returns the pruned classification tree again as a data frame, which can be converted to a 'party' object by the two main functions. The columns of information contained in the accepted tree argument and the final returned tree are described in Section 4.6.

4.5. Details About Argument Inputs

For both the `dipm()` and `spmtree()` functions, consider the following requirements for argument inputs. For the `formula` argument, the formula must take format `Y ~ treatment | X1 + X2` for data with a continuous outcome variable Y and `Surv(Y, δ) ~ treatment | X1 + X2` for data with a survival outcome variable Y and a status indicator δ. A format such as `Y ~ treatment | .` may be used when all variables in the data, excluding Y, δ (if applicable), and the treatment variable, are to be used as candidate split variables. Note that this notation is borrowed from the formats used in the **partykit** (Seibold *et al.* 2016; Zeileis *et al.* 2008) and **quint** (Dusseldorp and Van Mechelen 2014) R packages for consistency.

For the `data` argument, the supplied dataset must contain an outcome variable Y and a treatment variable. If Y is a right-censored survival time outcome, then there must also be a status indicator δ, where values of 1 denote the occurrence of the (harmful) event of interest, and values of 0 denote censoring. If there are only two treatment groups, then the two possible values must be 0 or 1. If there are more than two treatment groups, then the possible values must be integers starting from 1 to the total number of treatment assignments. In regard to the candidate split variables, if a variable is binary, then the variable must take values of 0 or 1. If a variable is nominal, then the values must be integers starting from 1 to the total number of categories. There cannot be any missing values in the dataset. For candidate split variables with missing values, the missings together (MT) method proposed by Zhang, Holford, and Bracken (1996) is helpful.

For the `types` argument, the default is to assume all of the candidate split variables are ordinal. If this is not the case, then all of the variables in the data must be specified with a vector of characters in the order that the variables appear. The possible variable types are: "binary", "ordinal", "nominal", "response", "status", and "treatment" for binary candidate split variables, ordinal candidate split variables, nominal candidate split variables, outcome variable Y, the status indicator δ (if applicable), and the treatment variable respectively.

4.6. Details About Returned Output

For the `dipm()` and `spmtree()` functions, the final classification tree is returned as a 'party' object by default. The option of returning the tree as a data frame is also provided. For the `pmprune()` function, the final classification tree is returned as a data frame. The returned data frame contains the following columns of information:

- `node`: Unique integer values that identify each node in the tree, where all of the nodes are indexed starting from 1.
- `splitvar`: Integers that represent the candidate split variable used to split each node, where all of the variables are indexed starting from 1.
- `splitvar_name`: The names of the candidate split variables used to split each node obtained from the column names of the supplied data.

- `type`: Characters that denote the type of each candidate split variable. "bin" denotes binary variables, "ord" denotes ordinal, and "nom" denotes nominal.
- `splitval`: Values of the left child node of the current split/node. For binary variables, a value of 0 is printed, and subjects with values of 0 for the current `splitvar` are in the left child node, while subjects with values of 1 are in the right child node. For ordinal variables, `splitval` is numeric and implies that subjects with values of the current `splitvar` less than or equal to `splitval` are in the left child node, while the remaining subjects with values greater than `splitval` are in the right child node. For nominal variables, the `splitval` is a set of integers separated by commas, and subjects in that set of categories are in the left child node, while the remaining subjects are in the right child node.
- `lchild`: Integers that represent the index (i.e., `node` value) of each node's left child node.
- `rchild`: Integers that represent the index (i.e., `node` value) of each node's right child node.
- `depth`: Integers that specify the depth of each node. The root node has depth 1, its children have depth 2, etc.
- `nsubj`: Integers that count the total number of subjects within each node.
- `besttrt`: Integers that denote the identified best treatment assignment of each node.

Note that for columns `splitvar`, `splitvar_name`, `type`, `splitval`, `lchild`, and `rchild`, for terminal nodes, i.e., nodes without child nodes, their values are set equal to `NA`.

5. Illustrative Examples

5.1. A Continuous Outcome Variable and Two Treatment Groups

Consider an example dataset with a continuous outcome variable Y, two treatment groups, and a sample size of 300. There are 5 candidate split variables that are all ordinal and independent and normally distributed, i.e., N(0, 1), named X_1, X_2, ..., X_5. The underlying model for Y is a tree model of depth 3 in which X_1 is important at the first split, and X_2 and X_3 are important at the next two splits. For subjects with values of $X_1 \leq 0$ and $X_2 \leq 0$, treatment 0 is the optimal treatment. For subjects with values of $X_1 \leq 0$ and $X_2 > 0$, treatment 1 is the optimal treatment. For subjects with values of $X_1 > 0$ and $X_3 \leq 0$, treatment 0 is the optimal treatment. Finally, for subjects with values of $X_1 > 0$ and $X_3 > 0$, treatment 1 is the optimal treatment. The two treatment assignments are randomly generated with equal probability, i.e., Bin(300, 0.5). The R code used to generate these data is as follows:

```
R> N = 300
R> set.seed(123)
R>
```

```
R> # generate binary treatments
R> treatment = rbinom(N, 1, 0.5)
R>
R> # generate candidate split variables
R> X1 = rnorm(n = N, mean = 0, sd = 1)
R> X2 = rnorm(n = N, mean = 0, sd = 1)
R> X3 = rnorm(n = N, mean = 0, sd = 1)
R> X4 = rnorm(n = N, mean = 0, sd = 1)
R> X5 = rnorm(n = N, mean = 0, sd = 1)
R> X = cbind(X1, X2, X3, X4, X5)
R> colnames(X) = paste0("X", 1:5)
R>
R> # generate continuous outcome variable
R> calculateLink = function(X, treatment) {
+
+    ((X[, 1] <= 0) & (X[, 2] <= 0)) *
+    (25 * (1 - treatment) + 8 * treatment) +
+
+    ((X[, 1] <= 0) & (X[, 2] > 0)) *
+    (18 * (1 - treatment) + 20 * treatment) +
+
+    ((X[, 1] > 0) & (X[, 3] <= 0)) *
+    (20 * (1 - treatment) + 18 * treatment) +
+
+    ((X[, 1] > 0) & (X[, 3] > 0)) *
+    (8 * (1 - treatment) + 25 * treatment)
+ }
R>
R> Link = calculateLink(X, treatment)
R> Y = rnorm(N, mean = Link, sd = 1)
R>
R> # combine variables in a data frame
R> data = data.frame(X, Y, treatment)
```

Then, `dipm()` and `spmtree()` may both be used to construct a classification tree for these data. Suppose we are interested in creating each tree with a maximum depth of 3. For the DIPM tree, we use the version of the method that considers all possible splits of `mtry` = 2 of the candidate split variables at each node of embedded trees. Also, we use the default values for `types`, `ntree`, `nmin`, `nmin2`, `print`, `dataframe`, and `prune`. For the simple PM tree, we use the default values for `types`, `nmin`, `print`, `dataframe`, and `prune`.

```
R> tree1 = dipm(Y ~ treatment | ., data, mtry = 2, maxdepth = 3)

Note that all candidate split variables are assumed to be ordinal.

DIPM Tree (Continuous Y, 2 treatments, yes mtry, ntree=300):
node splitvar splitvar_name type     splitval lchild rchild depth nsubj besttrt
```

```
   1        1             X1  ord -0.014307413      2      3     1   300       1
   2        2             X2  ord -0.016002527      4      5     2   148       0
   3        3             X3  ord -0.004060653      6      7     2   152       1
   4       NA           <NA> <NA>           NA     NA     NA     3    68       0
   5       NA           <NA> <NA>           NA     NA     NA     3    80       1
   6       NA           <NA> <NA>           NA     NA     NA     3    71       0
   7       NA           <NA> <NA>           NA     NA     NA     3    81       1
```

```
R> tree2 = spmtree(Y ~ treatment | ., data, maxdepth = 3)
```

```
Note that all candidate split variables are assumed to be ordinal.

SPM Tree (Continuous Y, 2 treatments):
node splitvar splitvar_name type     splitval lchild rchild depth nsubj besttrt
   1        1            X1  ord -0.014307413      2      3     1   300       1
   2        2            X2  ord -0.016002527      4      5     2   148       0
   3        3            X3  ord -0.004060653      6      7     2   152       1
   4       NA          <NA> <NA>           NA     NA     NA     3    68       0
   5       NA          <NA> <NA>           NA     NA     NA     3    80       1
   6       NA          <NA> <NA>           NA     NA     NA     3    71       0
   7       NA          <NA> <NA>           NA     NA     NA     3    81       1
```

For these data, the two methods create identical trees. Since `types`, i.e., the types of each candidate split variable, are not specified, the method warns us that all of the candidate split variables are assumed to be ordinal. Also, since we use the default value of `TRUE` for `print`, each tree is automatically printed to the screen with a header describing the method used. To visualize each tree, we can plot the created objects to the screen:

```
R> plot(tree1, terminal_panel = node_dipm)
R> plot(tree2)
```

From Figure 2, we clearly see that X_1 is selected at the first split of each tree, and X_2 and X_3 are selected at the next two splits. The correct treatment assignments are also identified at the terminal nodes, i.e., nodes 3 through 7, of the trees. Figure 3 displays the tree using the default plot method provided by the **partykit** package. We can also use predict methods provided by the **partykit** package to predict responses (class '`constparty`') or terminal nodes (class '`party`') for new observations. Moreover, we can conveniently predict the optimal treatment option for new subjects by one line of code.

```
R> predict(as.constparty(tree1), newdata = head(data))
```

```
       1        2        3        4        5        6 
14.31105 18.85920 14.31105 19.19895 19.19895 17.61470 
```

```
R> predict(tree1, newdata = head(data))
```

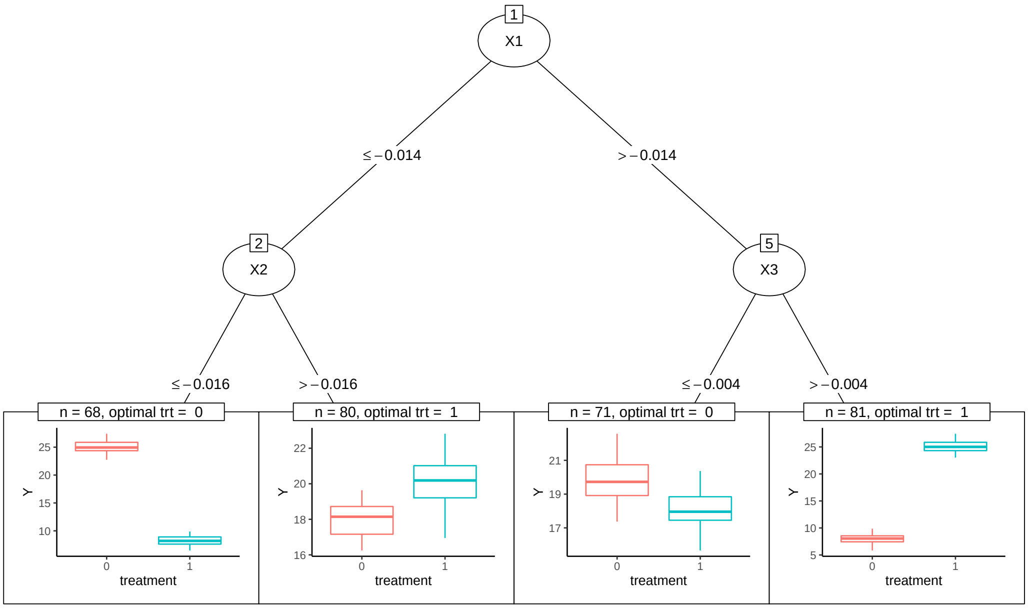

Figure 2: Tree visualization for the scenario of a continuous outcome and two treatment groups. The terminal nodes are visualized for comparing two treatments.

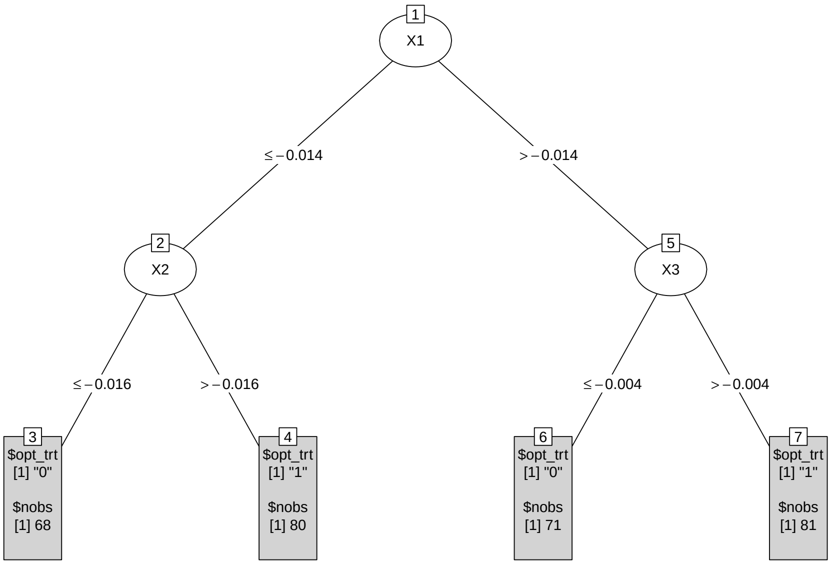

Figure 3: Tree visualization for the scenario of a continuous outcome and two treatment groups. The tree is plotted using the default plot method provided by the **partykit** package.

```
1 2 3 4 5 6
7 6 7 4 4 3

R> predict(tree1, newdata = head(data),
+    FUN = function(n) as.numeric(n$info$opt_trt))

1 2 3 4 5 6
1 0 1 1 1 0
```

5.2. A Continuous Outcome Variable and Three Treatment Groups

Next, consider a dataset with a continuous outcome variable Y, three treatment groups, and a sample size of 600. There are 7 candidate split variables named X_1, X_2, ..., X_7. X_1 and X_2 are ordinal and normally distributed, i.e., N(0, 1) rounded to the fourth decimal place. X_3 and X_4 are nominal and sampled from the discrete uniform distribution, i.e., $X_3 \sim$ Discrete Uniform[1, 4], and $X_4 \sim$ Discrete Uniform[1, 5]. The remaining three variables are binary, i.e., Bin(300, 0.5). The underlying model for Y is linear in which X_1 is the only important variable. For subjects with larger values of X_1, treatment 1 is the optimal treatment. The three treatment assignments are randomly generated with equal probability, i.e., Discrete Uniform[1, 3]. The R code used to generate these data is as follows:

```
R> N = 600
R> set.seed(123)
R>
R> # generate treatments
R> treatment = sample(1:3, N, replace = TRUE)
R>
R> # generate candidate split variables
R> X1 = round(rnorm(n = N, mean = 0, sd = 1), 4)
R> X2 = round(rnorm(n = N, mean = 0, sd = 1), 4)
R> X3 = sample(1:4, N, replace = TRUE)
R> X4 = sample(1:5, N, replace = TRUE)
R> X5 = rbinom(N, 1, 0.5)
R> X6 = rbinom(N, 1, 0.5)
R> X7 = rbinom(N, 1, 0.5)
R> X = cbind(X1, X2, X3, X4, X5, X6, X7)
R> colnames(X) = paste0("X", 1:7)
R>
R> # generate continuous outcome variable
R> calculateLink = function(X, treatment) {
+
+    10.2 - 0.3 * (treatment == 1) - 0.1 * X[, 1] +
+    2.1 * (treatment == 1) * X[, 1] +
+    1.2 * X[, 2]
+ }
R>
```

```
R> Link = calculateLink(X, treatment)
R> Y = rnorm(N, mean = Link, sd = 1)
R>
R> # combine variables in a data frame
R> data = data.frame(X, Y, treatment)
R>
R> # create vector of variable types
R> types = c(rep("ordinal", 2), rep("nominal", 2), rep("binary", 3),
+    "response", "treatment")
```

Then, `dipm()` and `spmtree()` may both be used to construct a classification tree for these data. Suppose we are interested in creating a DIPM tree with a maximum depth of 2, and a simple PM tree with a maximum depth of 4. Since the candidate split variables are not all ordinal, we create a vector of variable types for the `types` argument in both trees. For the DIPM tree, we use the version of the method that considers all possible splits of all of the candidate split variables at each node of embedded trees. Also, we use the default values for `ntree`, `nmin`, `nmin2`, `print`, `dataframe`, and `prune`. For the simple PM tree, we use the default values for `nmin`, `print`, `dataframe`, and `prune`.

```
R> tree3 = dipm(Y ~ treatment | ., data, types = types, maxdepth = 2)

DIPM Tree (Continuous Y, 2+ treatments, no mtry, ntree=25):
node splitvar splitvar_name type splitval lchild rchild depth nsubj besttrt
   1        1            X1  ord   0.1464      2      3     1   600       2
   2       NA          <NA> <NA>       NA     NA     NA     2   325       3
   3       NA          <NA> <NA>       NA     NA     NA     2   275       1

R> tree4 = spmtree(Y ~ treatment | ., data, types = types, maxdepth = 4)

SPM Tree (Continuous Y, 2+ treatments):
node splitvar splitvar_name type splitval lchild rchild depth nsubj besttrt
   1        1            X1  ord   0.1464      2      3     1   600       2
   2        1            X1  ord  -0.6868      4      5     2   325       3
   3        1            X1  ord   1.5466      6      7     2   275       1
   4        3            X3  nom    {2,3}      8      9     3   145       2
   5        1            X1  ord   0.0261     10     11     3   180       3
   6        4            X4  nom    {4,5}     12     13     3   237       1
   7       NA          <NA> <NA>     <NA>     NA     NA     3    38       1
   8       NA          <NA> <NA>     <NA>     NA     NA     4    75       2
   9       NA          <NA> <NA>     <NA>     NA     NA     4    70       3
  10       NA          <NA> <NA>     <NA>     NA     NA     4   154       3
  11       NA          <NA> <NA>     <NA>     NA     NA     4    26       3
  12       NA          <NA> <NA>     <NA>     NA     NA     4    86       1
  13       NA          <NA> <NA>     <NA>     NA     NA     4   151       1
```

Since we use the default value of `TRUE` for `print`, each tree is automatically printed to the screen with a header describing the method used. The DIPM tree has only three nodes since

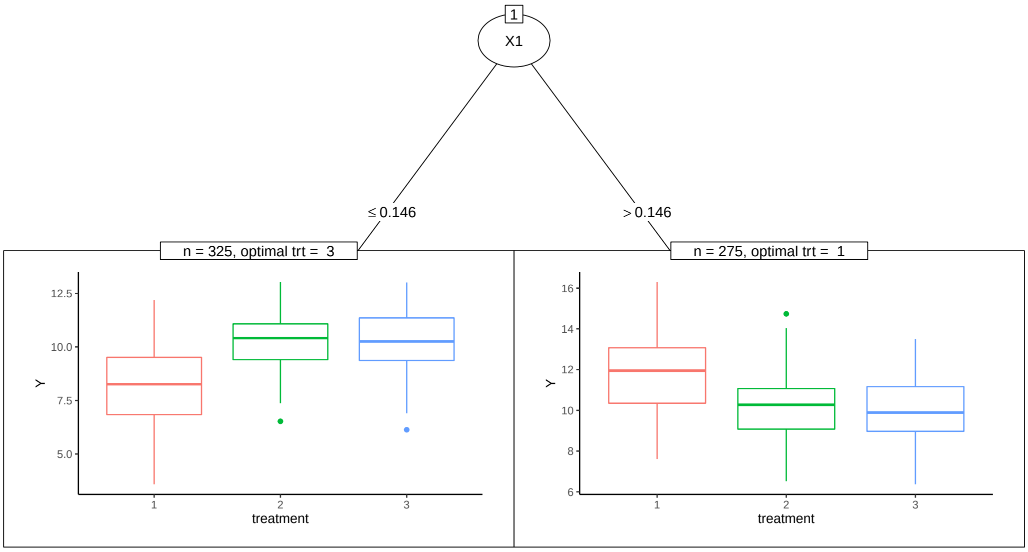

Figure 4: Tree visualization for the scenario of a continuous outcome and three treatment groups using the DIPM method.

we deliberately set the maximum tree depth to 2. Since we set a maximum depth of 4 for the simple PM tree, the tree is further grown. We may view the trees using the plot method. From Figure 4 and Figure 5, we see that X_1 is selected at the first split of each tree. As expected, treatment 1 is identified as the optimal treatment for subjects with larger values of X_1 in node 3 of Figure 4.

Finally, suppose we want to prune the simple PM tree which currently contains 13 total nodes. We may set the `prune` argument as `TRUE`:

```
R> ptree = spmtree(Y ~ treatment | ., data, types = types,
+    maxdepth = 4, prune = TRUE)
```

From Figure 6, we can see that terminal node pairs with the same recommended best treatment assignment are removed, and the pruned tree contains 7 nodes instead of 13.

5.3. Analysis of Anorexia Data

The weight change data for young female anorexia patients from the **MASS** package consists of 72 observations and 3 variables. Treat contains three treatment groups: "Cont" (control), "CBT" (cognitive behavioural treatment), and "FT" (family treatment). PreWeight is the weight in pounds of the patient before study period. Similarly, PostWeight represents that of the patient after study period. In our analysis, we consider PostWeight as the response of interest and fit a tree based on the DIPM method:

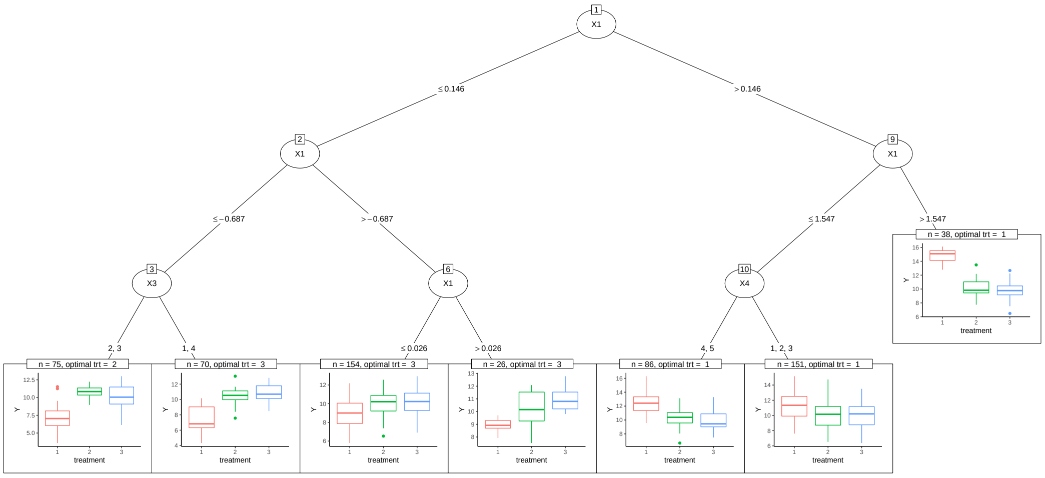

Figure 5: Tree visualization for the scenario of a continuous outcome and three treatment groups using the simple PM tree method.

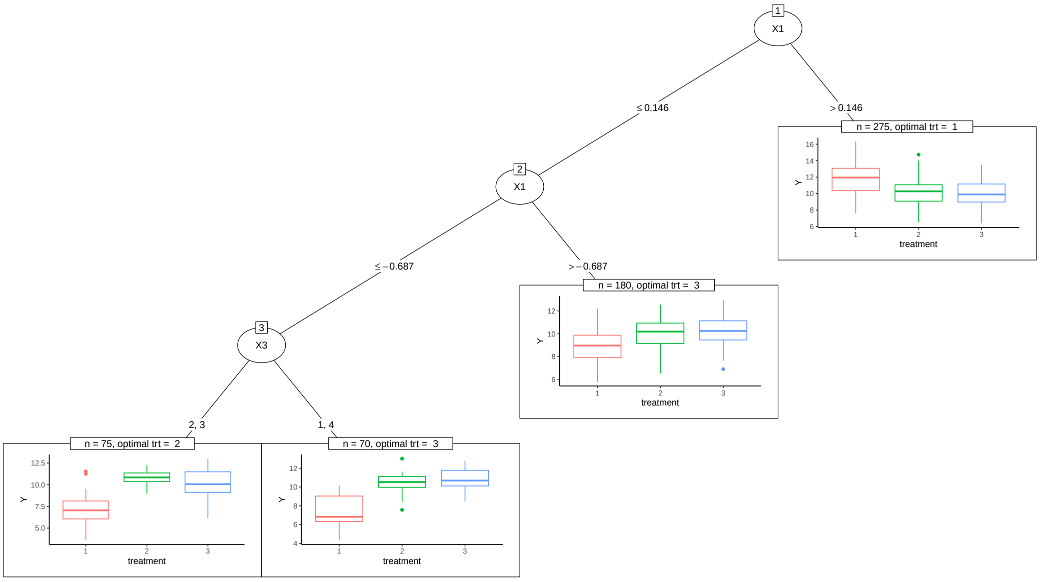

Figure 6: Tree visualization for the scenario of a continuous outcome and three treatment groups using the simple PM tree method. The tree is pruned.

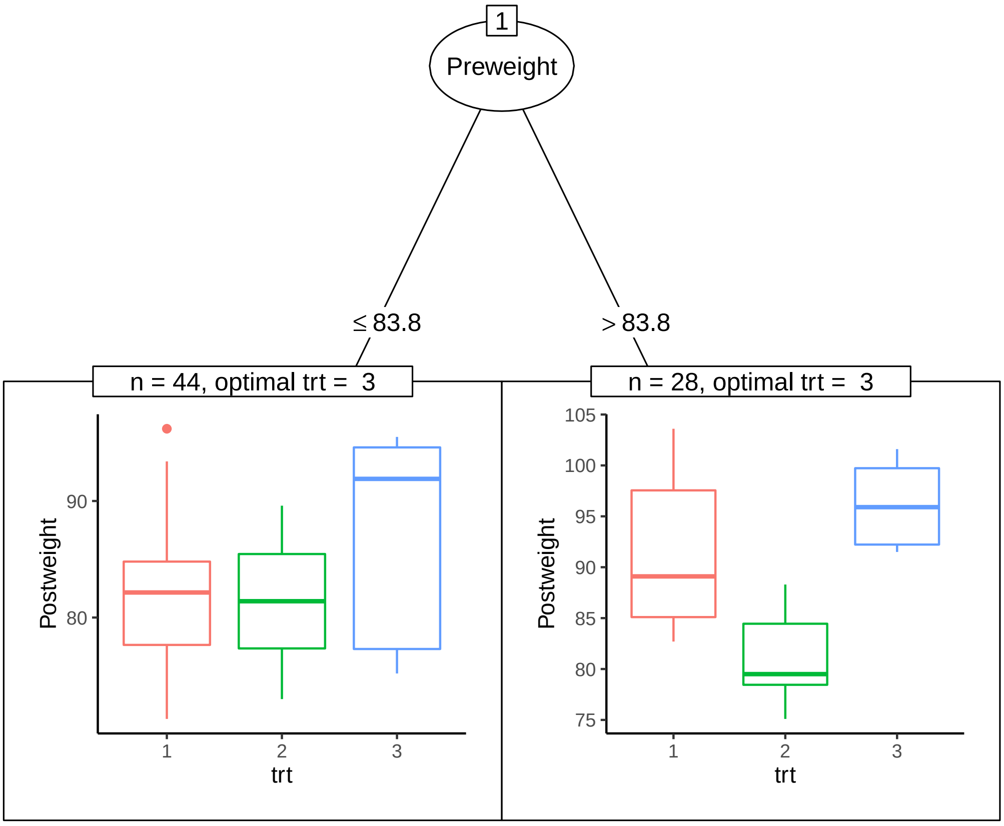

Figure 7: Tree visualization of anorexia data.

```
R> data("anorexia", package = "MASS")
R> data_anorexia = data.frame("Preweight" = anorexia$Prewt,
+    "Postweight" = anorexia$Postwt,
+    "trt" = as.integer(anorexia$Treat))
R> tree_anorexia = dipm(Postweight ~ trt | ., data_anorexia)
R> plot(tree_anorexia, terminal_panel = node_dipm)
```

Figure 7 visualizes the tree. For the patients with larger weights before the study, the effect of cognitive behavioural treatment is more profound compared with the control group. For all the patients, family treatment is the optimal treatment.

5.4. A Survival Outcome Variable and Two Treatment Groups

Consider a dataset with a right-censored survival outcome variable Y, two treatment groups, and a sample size of 300. There are 5 candidate split variables that are all ordinal and in-

dependent and normally distributed, i.e., N(0, 1), named X_1, X_2, ..., X_5. The underlying model for Y is an exponential model in which X_5 is the only important variable. For subjects with smaller values of X_5, treatment 1 is the optimal treatment. The two treatment assignments are randomly generated with equal probability, i.e., Bin(300, 0.5). The R code used to generate these data is as follows:

```
R> N = 300
R> set.seed(321)
R>
R> # generate binary treatments
R> treatment = rbinom(N, 1, 0.5)
R>
R> # generate candidate split variables
R> X1 = rnorm(n = N, mean = 0, sd = 1)
R> X2 = rnorm(n = N, mean = 0, sd = 1)
R> X3 = rnorm(n = N, mean = 0, sd = 1)
R> X4 = rnorm(n = N, mean = 0, sd = 1)
R> X5 = rnorm(n = N, mean = 0, sd = 1)
R> X = cbind(X1, X2, X3, X4, X5)
R> colnames(X) = paste0("X", 1:5)
R>
R> # generate survival outcome variable
R> calculateLink = function(X, treatment) {
+
+    X[, 1] + 0.5 * X[, 3] + (3 * treatment - 1.5) * (abs(X[, 5]) - 0.67)
+ }
R>
R> Link = calculateLink(X, treatment)
R> T = rexp(N, exp(-Link))
R> C0 = rexp(N, 0.1 * exp(X[, 5] + X[, 2]))
R> Y = pmin(T, C0)
R> delta = (T <= C0)
R>
R> # combine variables in a data frame
R> data = data.frame(X, Y, delta, treatment)
```

Next, `dipm()` and `spmtree()` may both be used to construct a classification tree for these data. Suppose we are interested in creating each tree with a maximum depth of 2. For the DIPM tree, we use the version of the method that considers all possible splits of all of the candidate split variables at each node of embedded trees and fit `ntree=1` tree at each node. Within each embedded tree, the maximum depth is set to 6. For the remaining arguments `types`, `nmin`, `nmin2`, `print`, `dataframe`, and `prune`, we use the default values. For the simple PM tree, we use the default values for `types`, `nmin`, `print`, `dataframe`, and `prune`.

```
R> tree5 = dipm(Surv(Y, delta) ~ treatment | ., data, ntree = 1,
+    maxdepth = 2, maxdepth2 = 6)
```

```
Note that all candidate split variables are assumed to be ordinal.

DIPM Tree (Survival Y, 2 treatments, no mtry, ntree=1):
node splitvar splitvar_name type   splitval lchild rchild depth nsubj besttrt
   1        5            X5  ord -0.8333296      2      3     1   300       1
   2       NA          <NA> <NA>         NA     NA     NA     2    63       1
   3       NA          <NA> <NA>         NA     NA     NA     2   237       0
```

```
R> tree6 = spmtree(Surv(Y, delta) ~ treatment | ., data, maxdepth = 2)
```

```
Note that all candidate split variables are assumed to be ordinal.

SPM Tree (Survival Y, 2 treatments):
node splitvar splitvar_name type   splitval lchild rchild depth nsubj besttrt
   1        5            X5  ord -0.8333296      2      3     1   300       1
   2       NA          <NA> <NA>         NA     NA     NA     2    63       1
   3       NA          <NA> <NA>         NA     NA     NA     2   237       0
```

For these data, the two methods create identical trees. Since `types`, i.e., the types of each candidate split variable, are not specified, the method warns us that all of the candidate split variables are assumed to be ordinal. Also, since we use the default value of `TRUE` for `print`, each tree is automatically printed to the screen with a header describing the method used. To view the full structure for each tree, we can plot the created objects:

```
R> plot(tree5, terminal_panel = node_dipm)
```

From Figure 8, we can see that X_5 is selected at the first split of each tree. As expected, treatment 1 is identified as the optimal treatment for subjects with smaller values of X_5 in node 2.

Responses, nodes, and optimal treatments for new subjects can be predicted as before.

```
R> predict(as.constparty(tree5), newdata = head(data))

        1         2         3         4         5         6
0.6164284 0.6164284 0.6164284 1.0854037 0.6164284 0.6164284
```

```
R> predict(tree5, newdata = head(data))

1 2 3 4 5 6
3 3 3 2 3 3
```

```
R> predict(tree5, newdata = head(data),
+    FUN = function(n) as.numeric(n$info$opt_trt))

1 2 3 4 5 6
0 0 0 1 0 0
```

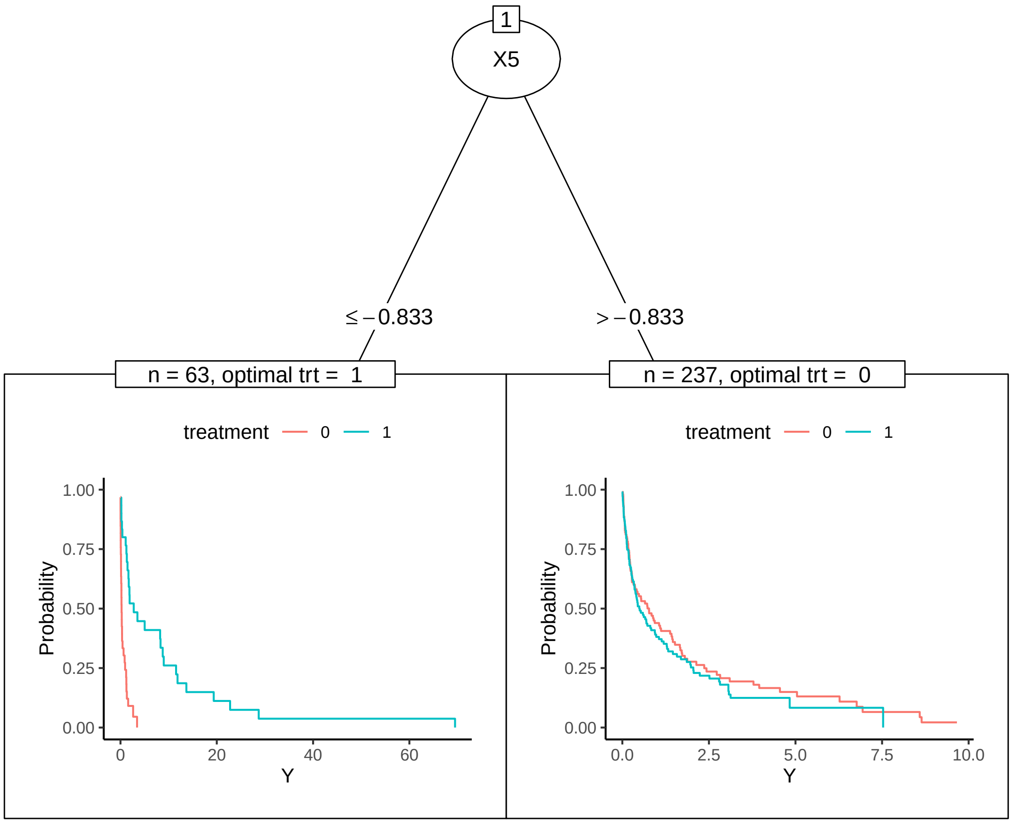

Figure 8: Tree visualization for the scenario of a survival outcome and two treatment groups.

5.5. A Survival Outcome Variable and Four Treatment Groups

Consider a dataset with a right-censored survival outcome variable Y, four treatment groups, and a sample size of 800. There are 7 candidate split variables named X_1, X_2, ..., X_7. X_1 and X_2 are ordinal and normally distributed, i.e., N(0, 1) rounded to the fourth decimal place. X_3 and X_4 are nominal and sampled from the discrete uniform distribution, i.e., $X_3 \sim$ Discrete Uniform[1, 4], and $X_4 \sim$ Discrete Uniform[1, 5]. The remaining three variables are binary, i.e., Bin(300, 0.5). The underlying model for Y is from the Weibull distribution in which X_1 is the only important variable. For subjects with larger values of X_1, treatment 1 is the optimal treatment. The four treatment assignments are randomly generated with equal probability, i.e., Discrete Uniform[1, 4]. The R code used to generate these data is as follows:

```
R> N = 800
R> set.seed(321)
R>
R> # generate treatments
R> treatment = sample(1:4, N, replace = TRUE)
R>
R> # generate candidate split variables
R> X1 = round(rnorm(n = N, mean = 0, sd = 1), 4)
R> X2 = round(rnorm(n = N, mean = 0, sd = 1), 4)
R> X3 = sample(1:4, N, replace = TRUE)
R> X4 = sample(1:5, N, replace = TRUE)
R> X5 = rbinom(N, 1, 0.5)
R> X6 = rbinom(N, 1, 0.5)
R> X7 = rbinom(N, 1, 0.5)
R> X = cbind(X1, X2, X3, X4, X5, X6, X7)
R> colnames(X) = paste0("X", 1:7)
R>
R> # generate survival outcome variable
R> calculateLink = function(X, treatment, noise) {
+
+    -0.2 * (treatment == 1) +
+    -1.1 * X[, 1] +
+    1.2 * (treatment == 1) * X[, 1] +
+    1.2 * X[, 2]
+ }
R>
R> Link = calculateLink(X, treatment)
R> T = rweibull(N, shape = 2, scale = exp(Link))
R> Cnoise = runif(n = N) + runif(n = N)
R> C0 = rexp(N, exp(0.3 * -Cnoise))
R> Y = pmin(T, C0)
R> delta = (T <= C0)
R>
R> # combine variables in a data frame
R> data = data.frame(X, Y, delta, treatment)
```

```
R>
R> # create vector of variable types
R> types = c(rep("ordinal", 2), rep("nominal", 2), rep("binary", 3),
+    "response", "status", "treatment")
```

dipm() and spmtree() may both be used to construct a classification tree for these data. Suppose we are interested in trees with a maximum depth of 2. Since the candidate split variables are not all ordinal, we create a vector of variable types for the types argument in each tree. For the DIPM tree, we use the version of the method that considers all possible splits of all of the candidate split variables at each node of embedded trees. One embedded tree is fit in the forest at each node, and the maximum depth of embedded trees is 6. For the remaining arguments nmin, nmin2, print, dataframe, and prune, the default values are used. For the simple PM tree method, we create two trees. The first tree uses all of the candidate split variables X_1 through X_7. The second tree only uses X_3 and X_4. Both trees use the default values for nmin, print, dataframe, and prune.

```
R> tree7 = dipm(Surv(Y, delta) ~ treatment | ., data,
+    types = types, ntree = 1, maxdepth = 2, maxdepth2 = 6)

DIPM Tree (Survival Y, 2+ treatments, no mtry, ntree=1):
node splitvar splitvar_name type splitval lchild rchild depth nsubj besttrt
   1        1            X1  ord   0.4154      2      3     1   800       4
   2       NA          <NA> <NA>       NA     NA     NA     2   524       3
   3       NA          <NA> <NA>       NA     NA     NA     2   276       1

R> tree8 = spmtree(Surv(Y, delta) ~ treatment | ., data,
+    types = types, maxdepth = 2)

SPM Tree (Survival Y, 2+ treatments):
node splitvar splitvar_name type splitval lchild rchild depth nsubj besttrt
   1        1            X1  ord   0.4154      2      3     1   800       4
   2       NA          <NA> <NA>       NA     NA     NA     2   524       3
   3       NA          <NA> <NA>       NA     NA     NA     2   276       1

R> tree9 = spmtree(Surv(Y, delta) ~ treatment | X3 + X4, data,
+    types = types, maxdepth = 2)

SPM Tree (Survival Y, 2+ treatments):
node splitvar splitvar_name type splitval lchild rchild depth nsubj besttrt
   1        4            X4  nom    {2,5}      2      3     1   800       4
   2       NA          <NA> <NA>     <NA>     NA     NA     2   330       3
   3       NA          <NA> <NA>     <NA>     NA     NA     2   470       4
```

Since we use the default value of TRUE for print, each tree is automatically printed to the screen with a header describing the method used. We may plot the full structure for each tree.

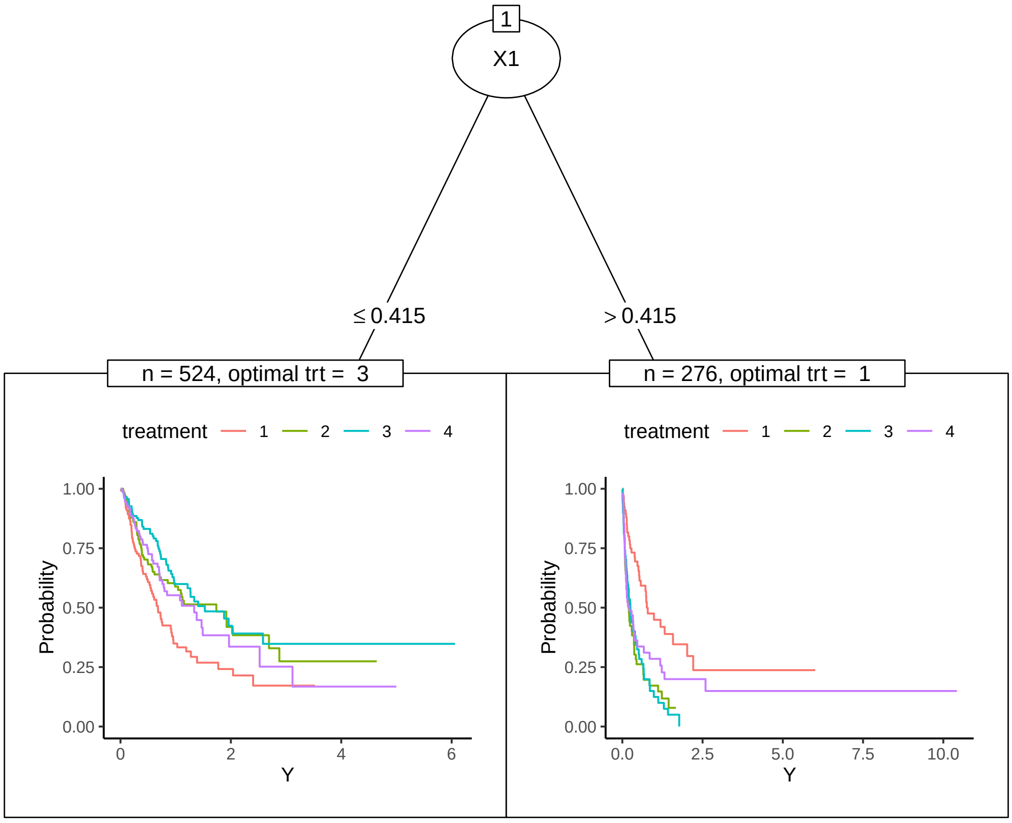

Figure 9: Tree visualization for the scenario of a survival outcome and four treatment groups using the DIPM method.

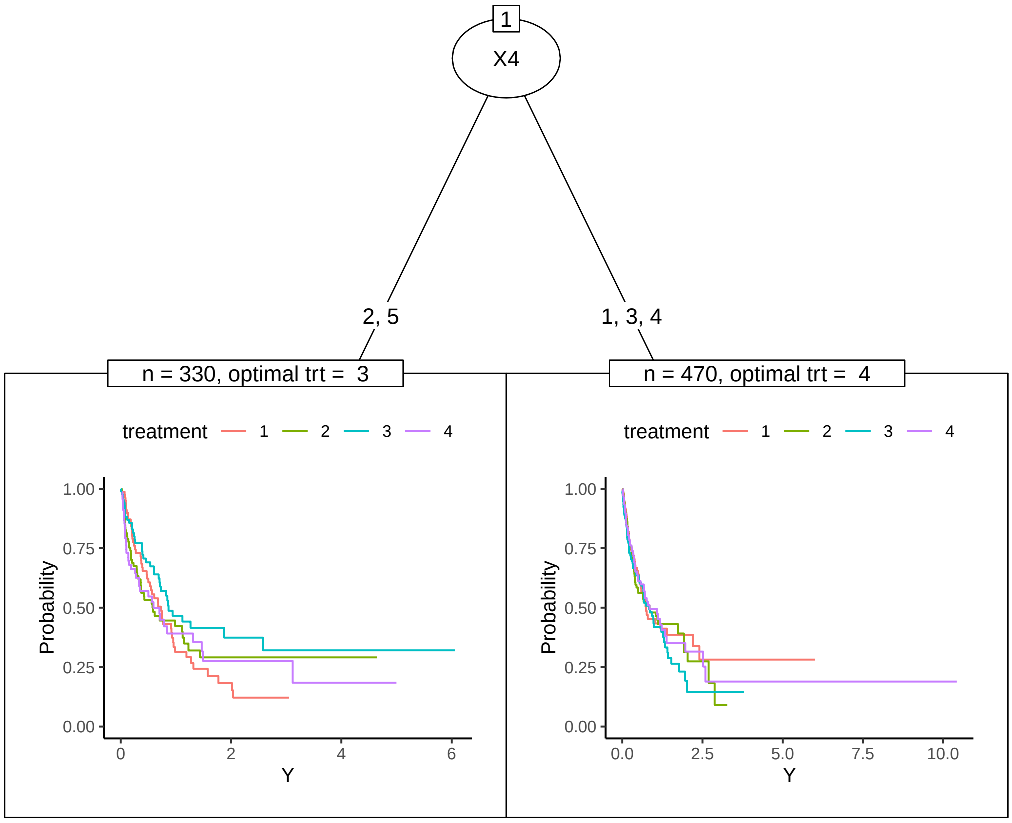

Figure 10: Tree visualization for the scenario of a survival outcome and four treatment groups using the simple PM tree method. Note that only two candidate split variables X_3 and X_4 are used.

For the DIPM tree and the first simple PM tree, Figure 9 shows that X_1 is selected at the first split. As expected, treatment 1 is identified as the optimal treatment for subjects with larger values of X_1 in node 3. However, Figure 10 shows that in the second simple PM tree, out of the only two candidate split variables used, X_3 and X_4, X_4 is selected. This example demonstrates that not all of the variables in the data have to be used as candidate split variables.

5.6. Analysis of Breast Cancer Data

The dataset from the package **TH.data** contains the observations of 686 women from the German Breast Cancer Study Group. The descriptions of the 10 variables are listed as follows: horTh represents the hormonal therapy (0 for no, 1 for yes); age is the age of the patients in years; menostat represents menopausal status, which is a factor at two levels premenopausal and postmenopausal; tsize is the tumor size in mm; tgrade represents tumor grade, which is a ordered factor at levels I < II < III; pnodes is number of positive nodes; progrec is progesterone receptor in fmol; estrec represents estrogen receptor in fmol; time is the recurrence free survival time in days; cens is a status indicator (0 for censored, 1 for event). We fit a survival tree based on the DIPM method:

```
R> data("GBSG2", package = "TH.data")
R> data_gbsg = data.frame("age" = GBSG2$age,
     "menostat" = as.integer(GBSG2$menostat == "Pre"),
     "tgrade" = as.integer(GBSG2$tgrade),
     "tsize" = GBSG2$tsize,
     "pnodes" = GBSG2$pnodes,
     "progrec" = GBSG2$progrec,
     "estrec" = GBSG2$estrec,
     "time" = GBSG2$time,
     "delta" = GBSG2$cens,
     "horTh" = as.integer(GBSG2$horTh) - 1)
R> types = c("ordinal", "binary", "nominal", rep("ordinal", 4),
+    "response", "status", "treatment")
R> tree_gbsg = dipm(Surv(time, delta) ~ horTh | ., data_gbsg,
+    types = types, ntree = 1, mtry = 2, maxdepth = 4)
R> plot(tree_gbsg, terminal_panel = node_dipm)
```

Figure 11 visualizes the tree. Hormonal therapy is most effective except for the patients with progesterone receptor less than 74 fmol and tumor grade I or progesterone receptor more than 74 fmol and tumor size greater than 26 mm and progesterone receptor greater than 320 fmol.

6. Discussion

In summary, the **dipm** R package implements the DIPM classification tree method designed for the analysis of clinical datasets with a continuous or right-censored survival outcome variable and two or more treatment groups. A secondary, additional method is also included in the package that employs a much simpler approach in identifying the best split at a node. Both

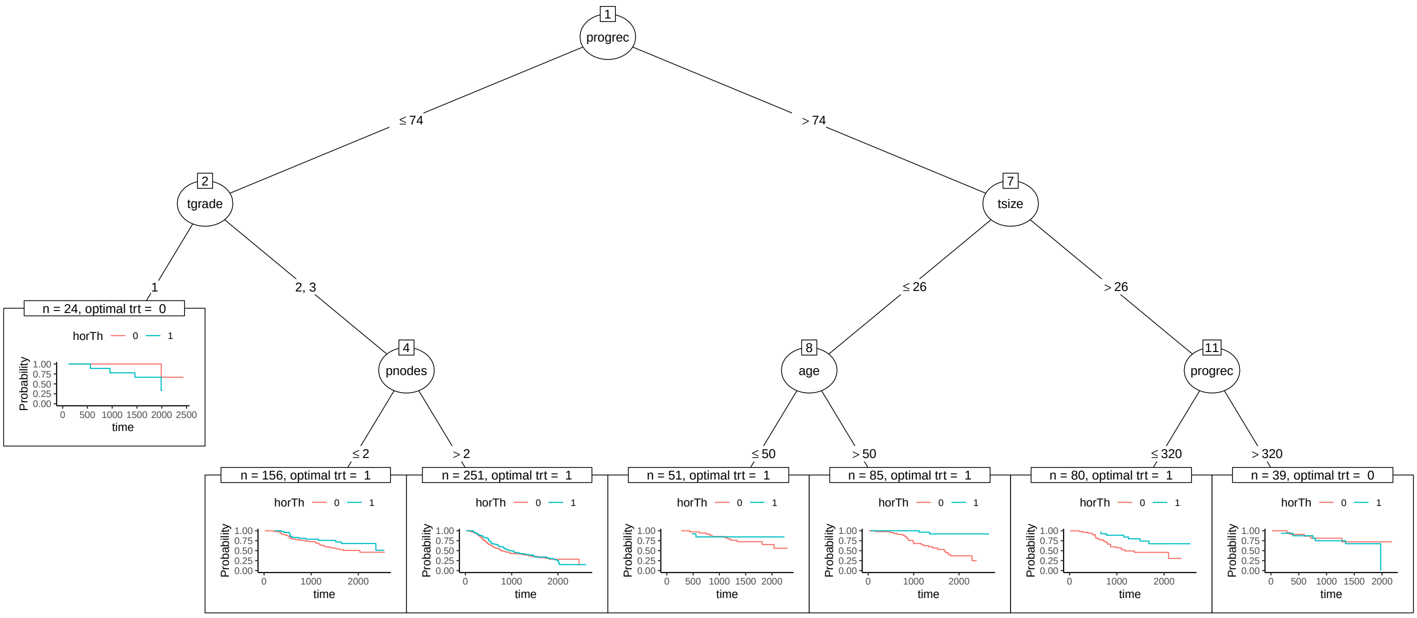

Figure 11: Tree visualization of breast cancer data.

methods have been evaluated in previous work (Chen and Zhang 2020, 2022). Real-world data applications are included and can also be found in previous papers. Furthermore, we add a plotting function that produces an image of each tree instead of solely a data frame of nodes. Overall, this vignette may be considered a user guide to implementing the DIPM method in the search for subgroups relevant to the precision medicine setting using datasets of one's own.

In the future, it would be useful to develop and implement a more sophisticated pruning strategy in comparison to the simple pruning approach proposed by Zhang *et al.* (2010) that we have already provided. Finally, because the DIPM method fits a forest of trees at each node and considers a broad pool of candidate splits at each node of the embedded trees, the method tends to take a longer time to run. Further improving computation time would be an important next step.

Acknowledgments

*These authors contributed equally to this work. This research is supported in part by NIH grants R01HG010171 and R01MH116527 and NSF grants DMS1722544 and DMS2112711.

References

Ashley EA (2016). "Towards Precision Medicine." *Nature Reviews Genetics*, **17**, 507–522.

Breiman L (2001). "Random Forests." *Machine Learning*, **45**, 5–32.

Chen V, Zhang H (2020). "Depth Importance in Precision Medicine (DIPM): A Tree and Forest Based Method." In *Contemporary Experimental Design, Multivariate Analysis and Data Mining*, pp. 243–259. Springer-Verlag.

Chen V, Zhang H (2022). "Depth importance in precision medicine (DIPM): a tree-and forest-based method for right-censored survival outcomes." *Biostatistics*, **23**(1), 157–172.

Chen X, Liu CT, Zhang M, Zhang H (2007). "A Forest-Based Approach to Identifying Gene and Gene-Gene Interactions." *Proceedings of the National Academy of Sciences of the United States of America*, **104**, 19199–19203.

Dusseldorp E, Doove L, van de Put J, Van Mechelen I (2018). ***quint**: Qualitative Interaction Trees.* R package version 2.0.0, URL https://CRAN.R-project.org/package=quint.

Dusseldorp E, Van Mechelen I (2014). "Qualitative Interaction Trees: A Tool to Identify Qualitative Treatment-Subgroup Interactions." *Statistics in Medicine*, **33**, 219–237.

Hothorn T, Hornik K, Zeileis A (2006). "Unbiased Recursive Partitioning: A Conditional Inference Framework." *Journal of Computational and Graphical Statistics*, **15**(3), 651–674.

Hothorn T, Zeileis A (2015). "**partykit**: A Modular Toolkit for Recursive Partytioning in R." *The Journal of Machine Learning Research*, **16**(1), 3905–3909.

Liaw A, Wiener M (2002). "Classification and Regression by **randomForest**." *R News*, **2**(3), 18–22. URL https://CRAN.R-project.org/doc/Rnews/.

Seibold H, Zeileis A, Hothorn T (2016). "Model-Based Recursive Partitioning for Subgroup Analyses." *International Journal of Biostatistics*, **12**, 45–63.

Seibold H, Zeileis A, Hothorn T (2019). "**model4you**: An R Package for Personalised Treatment Effect Estimation." *Journal of Open Research Software*, **7**(1).

Su X, Tsai CL, Wang H, Nickerson DM, Li B (2009). "Subgroup Analysis via Recursive Partitioning." *Journal of Machine Learning Research*, **10**, 141–158.

Therneau T, Atkinson B (2018). ***rpart**: Recursive Partitioning and Regression Trees.* R package version 4.1-13, URL https://CRAN.R-project.org/package=rpart.

Zeileis A, Hothorn T, Hornik K (2008). "Model-Based Recursive Partitioning." *Journal of Computational and Graphical Statistics*, **17**, 492–514.

Zhang H, Holford T, Bracken MB (1996). "A Tree-Based Method of Analysis for Prospective Studies." *Statistics in Medicine*, **15**, 37–49.

Zhang H, Legro RS, Zhang J, Zhang L, Chen X, Huang H, Casson PR, Schlaff WD, Diamond MP, Krawetz SA, Coutifaris C, Brzyski RG, Christman GM, Santoro N, Eisenberg E (2010). "Decision Trees for Identifying Predictors of Treatment Effectiveness in Clinical Trials and its Application to Ovulation in a Study of Women with Polycystic Ovary Syndrome." *Human Reproduction*, **25**, 2612–2621.

Zhu R, Zhao YQ, Chen G, Ma S, Zhao H (2017). "Greedy Outcome Weighted Tree Learning of Optimal Personalized Treatment Rules." *Biometrics*, **73**, 391–400.

Affiliation:

Victoria Chen
Department of Biostatistics
Yale School of Public Health
E-mail: victoria.chen@yale.edu

Cai Li
Department of Biostatistics
St. Jude Children's Research Hospital
E-mail: cai.li@stjude.org

Heping Zhang
Department of Biostatistics
Yale School of Public Health
E-mail: heping.zhang@yale.edu
